# Characterization of Diarrheagenic Enteroaggregative *Escherichia coli* in Danish Adults—Antibiotic Treatment Does Not Reduce Duration of Diarrhea

**DOI:** 10.3389/fcimb.2018.00306

**Published:** 2018-09-27

**Authors:** Betina Hebbelstrup Jensen, Camilla Adler Sørensen, Stig Hebbelstrup Rye Rasmussen, Dorthe Rejkjær Holm, Alice Friis-Møller, Jørgen Engberg, Hengameh C. Mirsepasi-Lauridsen, Carsten Struve, Anette M. Hammerum, Lone Jannok Porsbo, Randi Føns Petersen, Andreas Munk Petersen, Karen Angeliki Krogfelt

**Affiliations:** ^1^Department of Bacteria, Parasites & Fungi, Statens Serum Institut, Copenhagen, Denmark; ^2^The Research Unit for General Practice and Section of General Practice, Department of Public Health, University of Copenhagen, Copenhagen, Denmark; ^3^Department of Political Sciences and Public Management, University of Southern Denmark, Odense, Denmark; ^4^Department of Clinical Microbiology, Hvidovre Hospital, Copenhagen, Denmark; ^5^Department of Clinical Microbiology, Slagelse Hospital, Slagelse, Denmark; ^6^National Food Institute, Technical University of Denmark, Copenhagen, Denmark; ^7^Department of Virus & Microbiological Special Diagnostics, Statens Serum Institut, Copenhagen, Denmark; ^8^Department of Gastroenterology, Hvidovre Hospital, Copenhagen, Denmark

**Keywords:** Enteroaggregative *Escherichia coli*, EAEC, diarrhea, antibiotic resistance, multidrug resistance

## Abstract

Enteroaggregative *Escherichia coli* (EAEC) is frequently isolated from sporadic cases of diarrhea and in outbreaks of gastroenteritis in several regions of the world. The pathophysiology of EAEC continues to be enigmatic, and the efficacy of antibiotic treatment in EAEC-associated diarrhea has been discussed. Since the level of antibiotic resistance is increasing, it is essential to restrict the use of antibiotics to prevent further resistance development. We aimed to investigate EAEC strains in adult Danish patients suffering from diarrhea and from healthy controls. We examined the antibiotic resistance in EAEC strains, the clinical response to antibiotic treatment in EAEC diarrheal cases, and the distribution of virulence genes in diarrheal cases. The EAEC strains were collected from patients suffering from diarrhea in a Danish multicenter study. A medical doctor interviewed the patients by using a questionnaire regarding gastrointestinal symptoms, exposures, and use of antibiotic and over-the-counter antidiarrheal drugs. Follow-up was performed after 3–5 months to inquire about differential diagnosis to gastrointestinal disease. A multiplex polymerase chain reaction characterized virulence genes in diarrheal cases. Finally, the level of antibiotic resistance was examined by using the disc diffusion method. Asymptomatic carriage of EAEC in the adult Danish population was rare, in contrast to findings in healthy Danish children. The duration of diarrhea was not shortened by antibiotic treatment, specifically ciprofloxacin treatment, or by over-the-counter antidiarrheal drugs. Follow-up revealed no pathology in diarrheal patients apart from irritable bowel syndrome in two patients. A high number of patients suffered from long-term diarrhea, which was associated with the enterotoxin EAST-1 and a high virulence factor score. A high level of antibiotic resistance was observed and 58% of the EAEC strains were multidrug resistant. Multidrug resistance was most pronounced in cases of travelers' diarrhea, and it was seen that antibiotic treatment did not reduce the duration of diarrhea.

## Introduction

Enteroaggregative *Escherichia coli* (EAEC) has been associated with persistent diarrhea in both children and adults (Bhan et al., 1989; Schultsz et al., 2000). However, the contributing EAEC virulence factors to this disease manifestation do not point out to one specific virulence factor. The EAEC has proved to consist of considerable genetic diversity and is believed to comprise pathogenic as well as nonpathogenic strains (Jenkins et al., 2006). A high proportion of asymptomatic carriage in children has been reported in several studies (Nataro et al., 2006; Nüesch-Inderbinen et al., 2013), yet very few studies have examined EAEC carriage in adults.

The EAEC is characterized by its aggregative adherence fimbriae (AAFs) that enable the bacteria to adhere to the intestinal epithelium and form a persistent biofilm. The EAEC is described as a very heterogeneous group with respect to its virulence genes and their regulation (Hebbelstrup Jensen et al., 2014).

The transcriptional activator AggR, involved in regulation of at least 44 genes, both plasmid-borne and chromosomally positioned, regulates expression of the AAF genes. The genes *aagR, aap* (codes for dispersin), and *aatA* (ABC transporter) are used for initial detection of EAEC, as is the chromosomal gene *aaiC*, which encodes a type VI secretion system (Sheikh et al., 2002; Dudley et al., 2006; Hebbelstrup Jensen et al., 2014). Nevertheless, there is no consensus regarding which EAEC genes are explicitly pathogenic.

Once adherence and biofilm formation are established, EAEC pathogenesis usually involves the release of toxins, and, particularly, serine protease autotransporters of the *Enterobacteriaceae* (SPATEs) are commonly found in EAEC strains. These include toxins such as Pet, Sat, SigA, SepA, and Pic, which can be involved in loosening of cellular tight junctions, mucosal damage, and hypersecretion, and promotion of biofilm formation (Navarro-García et al., 1999; Navarro-Garcia et al., 2010; Al-Hasani et al., 2000; Guyer et al., 2002; Coron et al., 2009).

The EAEC was originally characterized from Chilean children with diarrhea (Nataro et al., 1987), and is probably best known for its role in persistent childhood diarrhea in developing countries. However, EAEC is also commonly associated with travelers' diarrhea, and the English Intestinal Infectious Diseases Study recognized foreign travel as the risk factor most commonly associated with EAEC-related diarrhea in 2000 (Infectious Intestinal Disease Study Team, 2000; Mendez Arancibia et al., 2009; Hebbelstrup Jensen et al., 2014).

Antibiotic resistance and, especially, multidrug resistance (MDR) is of global concern, and the limitation of antibiotic usage is crucial. For EAEC, a high level of antibiotic resistance has been described previously (Sang et al., 1997; Khoshvaght et al., 2014; Hebbelstrup Jensen et al., 2016). Extended-spectrum beta-lactamase (ESBL) production and increased quinolone resistance have been detected in EAEC (Hebbelstrup Jensen et al., 2014). In most cases, EAEC infections are self-limiting, but, in persistent cases, antibiotic use can be deemed necessary. Glandt et al. investigated the clinical response to ciprofloxacin against a placebo treatment, and found that patients treated with ciprofloxacin had significant reductions in duration of diarrhea compared with controls (Glandt et al., 1999). This, and other studies, have suggested that fluoroquinolones, especially ciprofloxacin, may be the most effective antibiotic when treating EAEC infections (Cennimo et al., 2007). Unfortunately, resistance toward ciprofloxacin in EAEC strains has been reported in several studies (Hebbelstrup Jensen et al., 2014), and with the evidence of increasing resistance emerging in EAEC, in general, as well as other pathogens, it is important to investigate this development continuously.

In this study, we investigated EAEC in diarrheal cases, and determined its virulence in the Danish adult population. This is the first time EAEC strains are characterized in adult Danish patients and clinical symptoms are described. Furthermore, we looked into the effect of antibiotic treatment on the duration of EAEC-induced diarrhea, and determined the antibiotic resistance of the clinical EAEC isolates.

## Materials and methods

### Study population

Healthy controls: Asymptomatic carriage of EAEC in the gut of the Danish population was examined in stool samples collected from healthy individuals. Totally, 55 recruits submitted a stool sample and a questionnaire, including questions of antibiotic usage, in conjunction with a general health examination (Frøkjaer Jensen and Hammerum, 2008). The median age of the recruits was 33 years. Participation in the study was voluntary and written informed consent was required. An additional 103 healthy controls were recruited from employees and students from university settings in Copenhagen, where members of the research team explained the purpose of the study and received written informed consent prior to submission of a stool sample and a questionnaire. The exclusion criteria were use of antibiotics or reports of a diarrheal episode 14 days prior to stool sampling. All controls were screened for the presence of EAEC by polymerase chain reaction (PCR) targeting the genes *aatA, aggR*, and *aaiC*, as described in the section “EAEC Screening.”

Diarrheal cases: The study was conducted in the period from 2011 to 2014. The EAEC were investigated in stool samples from Danish patients with diarrhea, who had either visited their general practitioner (GP) or were hospitalized, and had submitted a stool sample for microbiological analysis in one of the three Departments of Clinical Microbiology (DCM) that participated in the study [Statens Serum Institut (SSI), Hvidovre Hospital, and Slagelse Hospital]. At Hvidovre Hospital and Slagelse Hospital, only patients suffering from travelers' diarrhea were investigated for the presence of EAEC. Therefore, to investigate the true prevalence of EAEC from all patients testing EAEC-positive, an assessment of the annual/seasonal distribution of EAEC in stool samples submitted for microbiological analysis for enteric pathogens at Statens Serum Institut was performed for the period between June 2011 and June 2012. At this unit, all categories of diarrhea were investigated for EAEC. A total of 436 patients, with a median age of 34 years, were included in the study. Patients suffering from diarrhea lasting longer than 90 days were excluded due to sampling at a late stage in diarrheal episodes and possible recall bias. To represent each patient, only one EAEC strain was included for analysis. Through questionnaires, the patients provided information concerning: duration and type of diarrhea, foreign travel within the previous 2 months, and the use of antibiotics or probiotics within the previous 14 days. The patients were interviewed by phone by a medical doctor or were contacted by e-mail using the same questionnaire. Follow-up was performed for a group of EAEC-positive patients, 3–6 months after initial contact, to assess their current health status and investigate if any possible differential diagnosis to diarrhea and gastrointestinal disease other than EAEC (e.g., inflammatory bowel disease, lactose intolerance, etc.) had emerged.

### Microbiological analysis

Stool samples underwent primary microbiological analysis at the participating Departments of Microbiology. This included microscopy for the enteric parasites *Blastocystis hominis, Giardia intestinalis*, and *Entamoeba* spp. and PCR for *Cryptosporidium* spp., *G. intestinalis, Entamoeba* spp., and *Dientamoeba fragilis*. Stool sample material was suspended in growth medium and cultured on the SSI selective enteric medium (Blom et al., 1999), and modified charcoal cefoperazone deoxycholate agar medium (SSI) (Hutchinson and Bolton, 1984), identifying *Salmonella* spp., *Campylobacter* spp., *Yersinia* spp., *Shigella* spp., *Vibrio* spp., *Cl. difficile, Aeromonas* spp., and diarrheagenic *E. coli*. At least five *E. coli* colonies showing different morphologies were screened by using PCR to identify the following *E. coli* pathotypes: EAEC, AEEC, ETEC, VTEC, EPEC, and EIEC (Persson et al., 2007; Boisen et al., 2012).

### EAEC screening

The EAEC strains were identified by targeting the genes *aatA, aggR*, and *aaiC* by multiplex PCR. Patients with EAEC-positive stool samples, in the age of 18 and older, who spoke Danish and lived in Denmark, were eligible for inclusion in this study. The EAEC stock cultures were frozen at −80°C in Luria-Bertani broth (LB, Sigma Aldrich) containing 10% (-vol/vol-) glycerol. Bacteria were cultivated in Dulbecco's Modified Eagle Medium containing 4.5 g/L d-Glucose (DMEM-HG, GibcoTM) for activation of virulence genes or LB for 16–18 h at 37°C, shaking at 180 rpm.

Initial identification for EAEC was performed at the Departments of Clinical Microbiology at Slagelse Hospital and Hvidovre Hospital by PCR targeting the *aggR* gene. The EAEC-positive strains were forwarded to the Danish National Reference Center for *Escherichia* and *Klebsiella* at SSI for further characterization as described later. To diagnose EAEC, the genes *aap* (dispersin protein), *aatA* (dispersin transporter protein), *aggR* (transcription activator), and *aaiC* (secreted protein) were targeted by PCR and DNA hybridization (Boisen et al., 2012). Detection of two of these genes was considered diagnostic of EAEC.

### Characterization of EAEC strains

For colony hybridization to detect genes *aggR, aatA*, and *aaiC*, bacteria were grown overnight on a blue agar plate (SSI) and then transferred to a Hybond-N+ nylon membrane (Amersham Pharmacia Biotech) placed on a heart infusion agar plate containing glucose (SSI). The digoxigenin-labeled probes had previously been described by Boisen et al. (2008) and the DNA-colony hybridization was modified from Struve et al. (2003).

Further characterization of the EAEC strains was performed by additional PCR, targeting the genes *sat, sepA, pic, sigA, pet, astA, aap, agg3/4C, agg3A, aafA, aggA, agg4A*, and *agg5A* as previously described (Boisen et al., 2012; Jønsson et al., 2015) with modifications. The characterization involved detection of the SPATEs including *sat* (secreted autotransporter toxin), *sepA* (*Shigella* extracellular protein), *pic* (serine protease precursor), *sigA* (IgA protease homolog), *pet* (plasmid encoded protein), and the *astA* gene, (EAEC heat-stable toxin). Furthermore, included in the characterization were *aap* (dispersin) and *aggA* (fimbrial subunit for AAF/I), *aafA* (fimbrial subunit for AAF/II), *agg3A* (fimbrial subunit for AAF/III), *agg3/4C* (usher for AAF/III-IV), *agg4A* (fimbrial subunit for AAF/IV), and *agg5A* (fimbrial subunit for AAF/V).

Multiplex (QIAGEN, Copenhagen, Denmark) and singleplex amplifications (Maxima Hot Start PCR master mix, Thermo Scientific Inc.) were performed according to the manufacturer's instructions, with annealing temperatures at 57°C and 1 min annealing. The PCR products were separated in 2% agarose gels and run for 1–1.5 h at 100 V.

### Susceptibility testing

Susceptibility toward antimicrobial agents was investigated by using the tablet diffusion method according to the Clinical and Laboratory Standards Institute, CLSI, guidelines [(Clinical Laboratory Standards Institute (CLSI), 2011)], where 0.5 McFarland standard on Müeller-Hinton II agar plates (BBLTM, US) were used. The EUCAST breakpoints (EUCAST.org, 2017) using the Neo-Sensitabs™ (Rosco Diagnostica A/S, Taastrup, Denmark) were used for the following agents: sulfamethoxazole 64 mg/L (ECOFF value), trimethoprim 2 mg/L, ciprofloxacin 0.064 mg/L, tetracycline 8 mg/L, meropenem 0.125 mg/L, azithromycin 8 mg/L, nalidixic acid16 mg/L, cefotaxime 0.25 mg/L, chloramphenicol 6 mg/L, tigecycline 0.5 mg/L, ceftazidime 0.5 mg/L, colistin 2 mg/L, ampicillin 8 mg/L, and gentamicin 2 mg/L. Multidrug resistance was defined as acquired resistance toward three or more antibiotics from different antibiotic classes tested (Magiorakos et al., 2012).

### Ethics

This study has been approved by The Danish National Committee on Health Research Ethics, protocol number [H-A-2009-066]. Written informed consent was obtained from the study participants. The study was approved by the Danish Data Protection Agency protocol number [2010-41-5405].

The Scientific Ethics Committee for the Copenhagen and Frederiksberg municipalities approved the protocol prior to the investigation of the recruits (KF 01-006/02).

### Statistics

We utilized the classification and regression tree (CART) Pro Version 6.0 (Salford Systems) software inputting factors of interest as binary (present/absent, yes/no) independent predictive variables along with a continuous “factor total” that was a sum of all factors. We performed a CART analysis using the likelihood ratio tests to identify statistically significant branching points between specific EAEC virulence genes and duration of diarrhea. We present a CART tree, where we treat it as a categorical construct distinguishing between short-term diarrhea (STD) (≤7 days) and long-term diarrhea (LTD) (≥14 days), respectively. Odds ratios were calculated for each individual EAEC gene in the group of adults with STD and LTD provided with a 95% confidence interval and *p*-values, by using Pearson X^2^. The T-tests and the multiple linear regression were performed in the statistical computing environment R (2017), R Core Team (2017), R: A language and environment for statistical computing, R Foundation for Statistical Computing, Vienna, Austria. URL https://www.R-project.org/.

## Results

### Study population

To investigate the rate of asymptomatic carriage of EAEC in the intestinal tract of the Danish population, we examined stool samples collected from 158 healthy individuals. Exclusion criteria were use of antibiotics or reports of a diarrheal episode 14 days prior to stool sampling. Only 2 healthy controls were found positive for EAEC (1.2%).

The annual prevalence of EAEC in patients with diarrhea was determined in a 1-year period from 2011 to 2012, where 10,036 diarrheal stool samples were submitted for microbiological examinations at SSI (no selection). The EAEC was detected in 470 (4.8%) of the stool samples, which accounted for ~4.6% of the patients.

**Figure 1:**
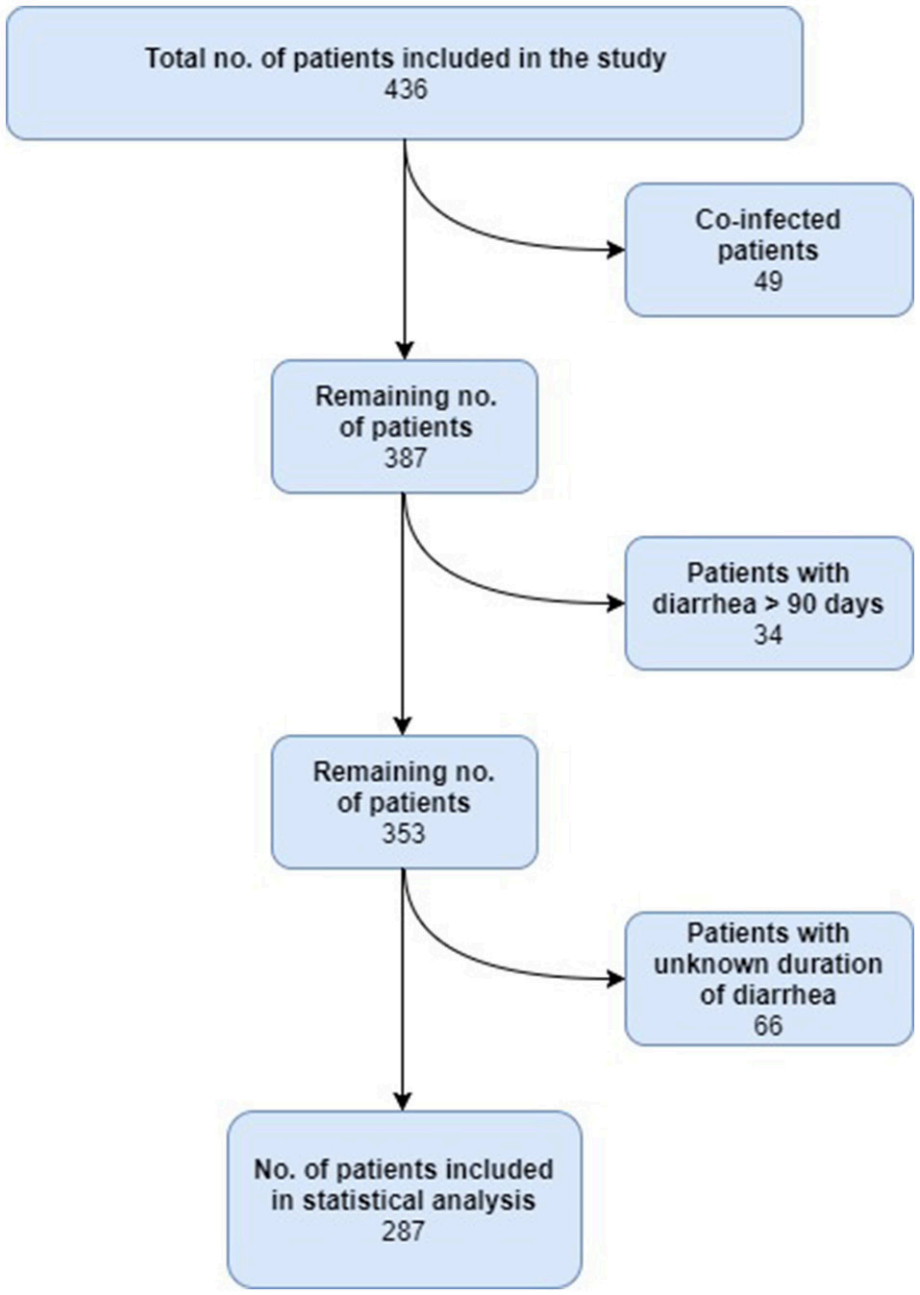
Flow diagram showing the patients included for analysis.

During the full study period from 2011 to 2014, 436 EAEC-positive patients were interviewed and deemed eligible for inclusion in the study. Between the 436 EAEC-positive patients, a total of 737 samples were collected at the participating units. In this study, only one sample was included per patient. In 49 patients, at least one other enteric pathogen was identified. The majority of the co-infections patients were seen to suffer from travelers' diarrhea (98%), and co-infections in this patient category has previously been described to occur frequently (Lääveri et al., 2014). The patients, who were co-infections, were excluded from further statistical analysis, as we wanted to investigate the clinical manifestations associated with EAEC only. Totally, 34 patients reported diarrheal duration to be higher than 90 days. These were excluded due to possible comorbidities and/or recall bias. Finally, 66 patients were excluded since the duration of diarrhea was unknown. A total of 287 patients were eligible for inclusion in the statistical analyses (Figure 1).

Follow-up was conducted after 3–5 months for 75 of the 436 EAEC-infected patients, where five patients had been diagnosed with gastrointestinal diseases prior to EAEC infection. Herein, two patients had Crohn's disease, two patients had irritable bowel disease, and one patient had ulcerative colitis. Within the follow-up period after EAEC infection, two patients were diagnosed with irritable bowel syndrome. Sixteen from the 76 patients available for follow-up had been referred to a specialist in gastroenterology. Of the 16 patients, 12 patients had undergone endoscopy to investigate the cause of diarrhea, with no signs of pathology a part from minor enteric inflammation.

### EAEC-associated symptoms

The median age of the 287 interviewed EAEC-positive patients was 34 years. The STD (≤7 days) was reported from 90 patients and intermediate duration of diarrhea (8–14 days) was reported from 83 patients; LTD lasting more than 14 days was reported from 114 patients. The episode of diarrhea had not ended in 51 patients at the time of the interview. The median duration of diarrhea was 14 days. Watery diarrhea was seen to be highly prevalent in patients infected with EAEC. In total, 256 patients (89%) reported watery diarrhea, 140 patients (49%) mucoid diarrhea, mixed watery, and mucoid diarrhea was reported in 140 patients, and 43 patients (15%) reported bloody diarrhea (Table 1). The median of the maximum number of diarrheal outputs per day was 7. Totally, 16 (6%) patients had been admitted to the hospital.

**Table 1:** Characteristics of EAEC-positive patients.

	**No. of patients**	**Median duration of diarrhea in days**	***p*-value**	**Confidence interval**
**POPULATION**				
Male	95	14	0.73	[−5.43; 3.80]
Female	192	14		
**DURATION OF DIARRHEA**				
Short-term (≤7 days)	90			
Intermediate (8–14 days)	83			
Long-term (>14 days)	114			
Ongoing	51			
**DIARRHEAL TYPE**				
Watery^£^	256	^Ω^	0.42^#^	[−3.58;8.35]
Mucoid	140		0.31	[−1.89;18.88]
Mixed watery and mucoid	140			
Bloody	43		0.22	[−1.50;6.60]
Travelers' diarrhea	265	14	0.38	[−5.31; 13.58]
Nontravelers' diarrhea	22	14		
Co-infection	49	10	0.00	[1.73;7.94]
No co-infection	287	14		
**USE OF ANTIBIOTICS**				
Yes	76	12	0.60	[−5.51;3.21]
No	186	14		
Not answered	25			
**USE OF ANTIBIOTICS**				
Ciprofloxacin				
Yes	32	14.5	0.86*^β^*	[−7.61;6.39]
No	320	13		
Penicillins	11			
Metronidazole	4			
Azithromycin	2			
Doxycycline	2			
Gentamycin	1			
Tazocin	1			
Tetracycline	1			
Nitrofurantoin	1			
Other	4			
Unknown	10			
Several antibiotics used	11			
**USE OF OTHER DIARRHEAL MEDICATION**				
Yes	129	14	0.22^#^	[−5.88; 1.34]
No	158	12		
Lopamide	68	14		
Probiotics				
Paraghurt	33	13		
Idoform	10	14.5		
Other	17	14		

£ *Several types of diarrhea were reported from the majority of the patients*.

Ω *The majority of diarrheal cases was mixed and the contribution to the duration of diarrhea could not be individualized*.

# *The p-values and confidence intervals for bloody, mucoid, and watery diarrhea are from a multiple regression using these three constructs as predictors of diarrhea length. The same analysis was performed for “Use of other diarrheal medication” compared with no usage of any medication*.

β *Patients with use of ciprofloxacin compared with patients without any use of antibiotics*.

### Travelers' diarrhea

The majority of the EAEC-positive patients suffered from travelers' diarrhea (*n* = 264, 92%). However, two of the participating units only investigated EAEC in cases of travelers' diarrhea. Various destinations were reported from the patients, who suffered from travelers' diarrhea. Totally, 59 patients had visited Egypt, 21 had been to Turkey, 31 to India, and 13 to Thailand. Tanzania, Germany, Cuba, and Nepal were each visited by 5 patients. Multiple travel destinations were reported from 57 patients. Several other travel destinations were reported, but with very few visits from patients. The duration of travelers' diarrhea was not seen to be longer compared with patients without reports of traveling (*p* = 0.38).

### Treatment of EAEC

Use of antibiotics was investigated as a predictor for the duration of EAEC-induced diarrhea. It was seen that the duration of diarrhea was not shortened by using antibiotics *p* = 0.6 (CI [−5.51; 3.21]). Since ciprofloxacin has been recommended for the treatment of EAEC (Glandt et al., 1999), we assessed the effect of antibiotics on diarrheal persistence. We did not observe a significant reduction of diarrhea by ciprofloxacin (*p* = 0.86, CI [−7.61; 6.39]). The usage of other antibiotics (median duration of diarrhea 13 days) was compared with the use of ciprofloxacin (median duration 14.5 days) and was not statistically significant, *p* = 0.53 (CI [−6.71; 3.53]). As a large part of the patients investigated suffered from traveler's diarrhea, it was investigated if the duration of EAEC-induced diarrhea was affected by consumption of probiotics and or use of other antidiarrheal drugs; no effects on the duration of diarrhea were seen from taking these over-the-counter remedies *p* = 0.22 (CI [−5.88; 1.34]) (Table 1).

### Co-infections

In our study, 49 patients (17%) were infected with EAEC and an additional microorganism. All, but one patient with co-infections was seen to suffer from travelers' diarrhea. The majority of the enteropathogens in co-infections were conventional microorganisms detected in cases of travelers' diarrhea. The AEEC was the most frequently detected pathogen in 18/49 EAEC cases with mixed pathogens, followed by ETEC, which was found among 13 EAEC cases. The highly prevalent enteropathogen in travelers' diarrhea *Campylobacter* spp. was found in 4 cases (Table 2). When different predictors were investigated for the duration of diarrhea, co-infections patients were seen to have a significant shorter duration compared with patients only infected with EAEC, *p* = 0.00.

**Table 2:** Distribution of enteropathogens in co-infections.

**Microorganisms in EAEC co-infections**	**Number of co-infected patients (*N* = 49)**
**BACTERIA**	
AEEC	18
ETEC	13
*Campylobacter* spp.	4
EPEC	2
*Aeromonas* spp.	2
VTEC	1
*Yersinia enterocolitica*	1
*Clostridium difficile*	1
*Shigella* spp.	1
**PARASITES**	
*Blastocystis hominis*	4
*Entamoeba* spp.	2
*Giardia lamblia*	2
*Cryptosporidium* spp.	1
**VIRUS**	
Norovirus	2

*From the 49 co-infected patients five were co-infected with several enteropathogens*.

### Distribution of EAEC virulence genes

Totally, 250 randomly selected stool samples of the 287, which had tested positive for the presence of *aggR* at either Hvidovre or Slagelse Hospital, were chosen for virulence gene characterization. However, by subsequent PCR and DNA hybridization, only 189 of the 250 samples were confirmed to have at least one of the EAEC genes tested for (*aggR, aatA, aaiC, aap*). In this study, EAEC was defined as testing positive for at least two of the previously mentioned four genes. Therefore, of the 189 isolates, 26 strains were excluded. Finally, nine isolates were excluded due to missing information in the questionnaires regarding duration of diarrhea. In total, 154 clinical EAEC isolates were eligible for further PCR characterization.

Assessing the prevalence of virulence genes in cases of STD and LTD, no significant differences in odds ratio between the two groups were observed. Table 3 presents the distribution of EAEC genes in strains isolated from adults with either STD or LTD (intermediate duration was not included, *n* = 19). In the 135 strains, the most abundant genes observed were *aggR* and *aap* at 97.8% (*n* = 132) and *aatA* at 88.9% (*n* = 120). The toxin gene most commonly found in the total number of strains was the Pic toxin (*pic*) present in 75.6% (*n* = 102) of the strains. The EAST1 toxin (*astA*) was observed in 34.8% (*n* = 47) of the total number of strains, and the sepA toxin (*sepA*) was found in 34.1% (*n* = 46) of the total number of strains. Of the fimbrial genes, AAF/I-AAF/V, AAF/I (*aggA*) was the most abundant in 22.2% (*n* = 30, total 135) followed by AAF/IV (*agg4A*) at 18.5%, AAF/III (*agg3A*) at 17%, AAF/V (*agg5A*) at 16.3%, and finally, AAF/II (*aafA*) at 12.6%.

**Table 3:** Distribution of EAEC virulence factors in cases of STD and LTD.

**EAEC**	**STD cases (*****n*** = **53)**		**LTD cases (*****n*** = **82)**		**Total (*****N*** = **135)**		
**Virulence gene**	**No. (%)**		**No. (%)**		**No. (%)**		**Odds Ratio**	**χ^2^ (Pearson)**	***P*-value (Pearson)**
*aatA*	48	(90.6)	72	(87.8)	120	(88.9)	0.75	0.25	0.62
*aggR*	52	(98.1)	80	(97.6)	132	(97.8)	0.77	–	–
*aaiC*	40	(75.5)	64	(78.0)	104	(77.0)	1.16	0.12	0.73
*aap*	52	(98.1)	80	(97.6)	132	(97.8)	0.77	–	–
*sat*	24	(45.3)	43	(52.4)	67	(49.6)	1.33	0.66	0.42
*sepA*	17	(32.1)	29	(35.4)	46	(34.1)	1.16	0.16	0.69
*pic*	39	(73.6)	63	(76.8)	102	(75.6)	1.19	0.18	0.67
*sigA*	3	(5.7)	8	(9.8)	11	(8.1)	1.80	-	-
*pet*	7	(13.2)	17	(20.7)	24	(17.8)	1.72	1.25	0.26
*astA*	14	(26.4)	33	(40.2)	47	(34.8)	1.88	2.71	0.10
*agg3/4C*	27	(50.9)	40	(48.8)	67	(49.6)	0.92	0.06	0.81
*agg5A*	9	(17.0)	13	(15.9)	22	(16.3)	0.92	0.03	0.86
*agg3A*	10	(18.9)	13	(15.9)	23	(17.0)	0.81	0.21	0.65
*aafA*	6	(11.3)	11	(13.4)	17	(12.6)	1.21	0.13	0.72
*aggA*	12	(22.6)	18	(22.0)	30	(22.2)	0.96	0.01	0.92
*agg4A*	9	(17.0)	16	(19.5)	25	(18.5)	1.19	0.14	0.71

*Isolates from 19 patients with intermediate duration of diarrhea were excluded, i.e., no belonging to either short-term diarrhea (STD) ≤7 days or long-term diarrhea (LTD) >14 days. However, the virulence profile of the isolates was determined and found similar to that of the other two (data not shown). p < 0.05 is significant*.

### CART analysis—assessing combinations of multiple EAEC virulence genes

**Figure 2:**
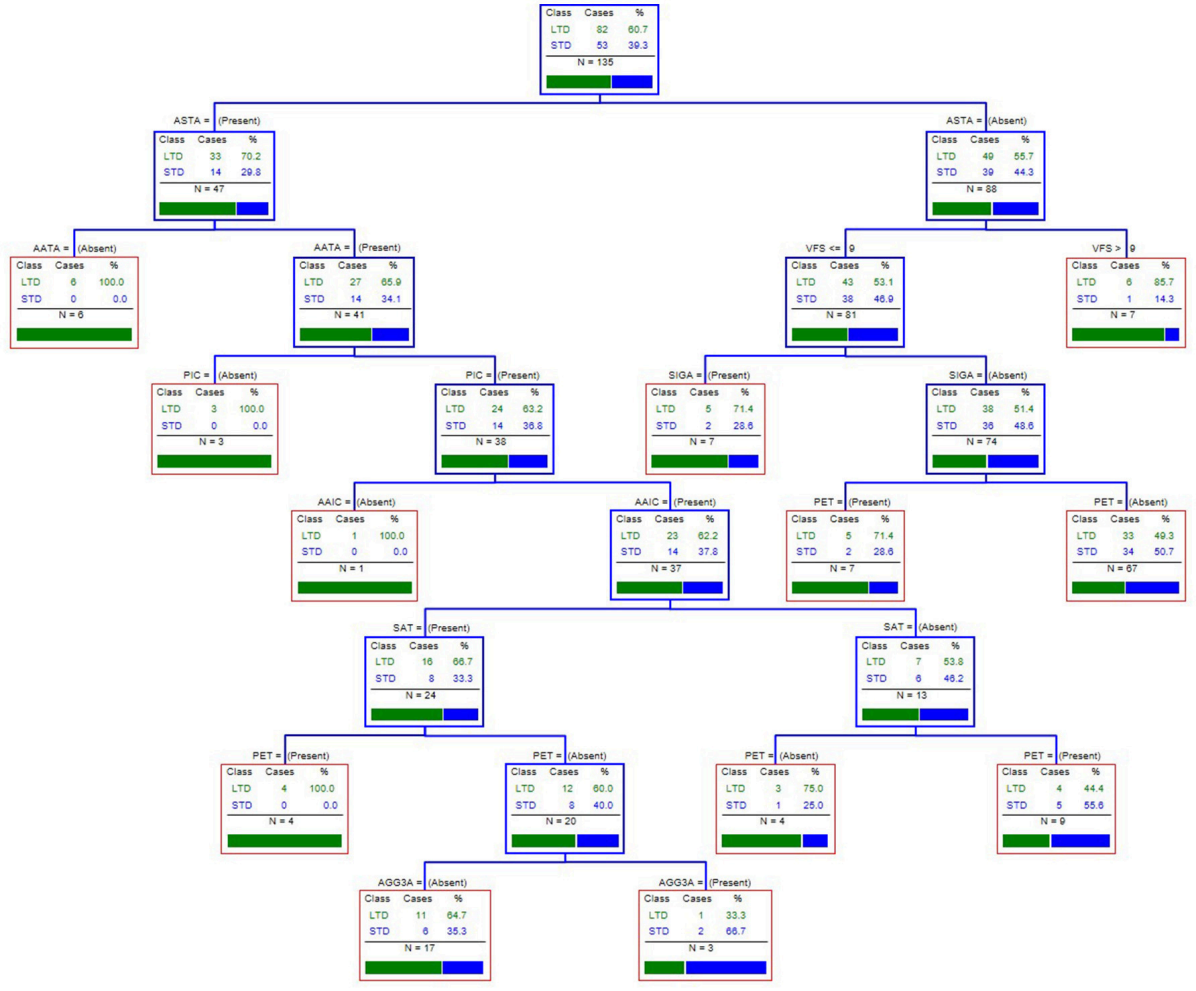
The CART tree analysis for assessing combinations of genotypic factors most strongly associated with long-term diarrhea. We considered all genotypic assays performed: *aatA, aggR, aaiC, aap, sat, sepA, pic, sigA, pet, astA, agg3/4C, agg5A, agg3A, aafA, aggA, agg4A*, and the virulence factor score (VFS). Each branch of the CART tree ends in a terminal node (red box), and each terminal node is defined by the presence or absence of a factor such as a gene or VFS. STD, short-term diarrhea; LTD, long-term diarrhea.

To consider the combination of virulence factors in association with STD and LTD, we performed a CART analysis. We considered all genotypic assays performed, and interrogated the association with the diarrheal duration. Of the 135 clinical EAEC isolates, 53 strains were isolated from adults with STD and 82 strains from adults with LTD. The CART analysis suggested that the presence of the *astA* gene, regardless of the presence or absence of any other scored genotype, showed an association with LTD. When lacking the *astA* gene, a high virulence factor score (VFS > 9) appeared to be associated with LTD, and if the VFS < 9, the toxin genes *sigA* and *pet* showed an association with LTD (Figure 2).

### Antibiotic resistance in EAEC isolates

**Figure 3:**
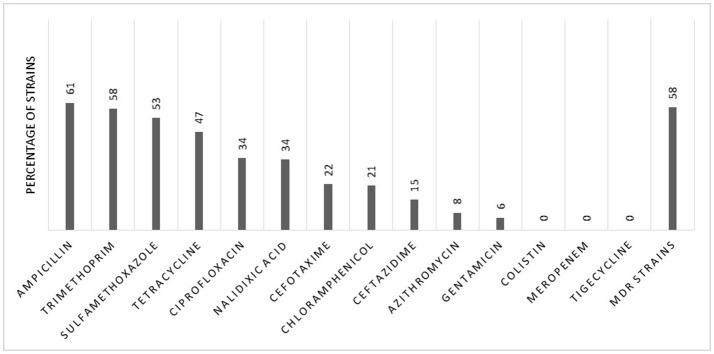
Patterns of antibiotic resistance in 137 clinical EAEC isolates.

Susceptibility testing toward antibiotics was performed for 137 of the 154 clinical EAEC isolates (17 isolates were not recovered). Overall, we observed a high level of resistance towards antibiotics (Figure 3). More than 50% of the strains were resistant toward ampicillin (*n* = 83) (61%), trimethoprim (*n* = 79) (58%), and sulfamethoxazole (*n* = 73) (53%), but high resistance levels were also observed for tetracycline (*n* = 64) (47%), ciprofloxacin (*n* = 47) (34%), and nalidixic acid (*n* = 46) (34%).

The MDR, defined as acquired resistance toward three or more antibiotics from different antibiotic classes tested, was observed in 58% (*n* = 80) of the strains (Figure 3). We observed no significant differences between the investigated parameters in non-MDR and MDR strains (Table 4).

**Table 4:** Characteristics of patients infected with antibiotic-resistant EAEC strains.

	**Non-MDR EAEC strains (*N* = 57)**	**MDR EAEC strains (*N* = 80)**	***p*-value^a^**
	**No. of patients (%)**	**No. of patients (%)**	
**DIARRHEAL DURATION**			
Short-term (≤7 days)	18 (32)	29 (36)	0.6
Intermediate (8–14 days)	6 (11)	9 (11)	1
Long-term (>14 days)	31 (54)	37 (46)	0.5
Unknown	2 (4)	5 (6)	–
**Antibiotic use**	15 (26)	23 (29)	0.8
Ciprofloxacin	10 (18)^*^	10 (13)^**^	0.5
Other	5 (9)	13 (16)	0.3
**Foreign travel**	50 (88)	70 (88)	1
**TRAVEL DESTINATIONS**			
Asia	19 (33)	34 (43)	0.3
Africa	12 (21)	24 (30)	0.3
Europe	6 (11)	2 (3)	0.07
South America	3 (5)	2 (3)	0.6
North America	2 (4)	–	–
Multiple destinations	9 (16)	8 (10)	0.5

a *Two-tailed Fisher Exact Probability Test*.

* *10/10 had reports of foreign travel*.

** *9/10 had reports of foreign travel*.

## Discussion

In our study, asymptomatic carriage of EAEC was rare, and from 158 healthy Danish adult controls, only two tested positive for EAEC (1.2%). We have previously shown a 10.5% carriage of EAEC in healthy Danish children (Hebbelstrup Jensen et al., 2016). A high number of healthy carriers of EAEC have been reported from studies conducted in children from the developing world (Kotloff et al., 2012). The high prevalence of asymptomatic carriage of EAEC in children has been explained by frequent exposure to EAEC and to acquired immunity in regions with reduced hygiene (Medina et al., 2010; Opintan et al., 2010). Our finding suggests that asymptomatic carriage of EAEC is not common in the Danish adult patients.

The duration of EAEC-induced diarrhea was seen to be shortened in patients, who were co-infections. As EAEC is generally considered to be noninvasive and of relatively low-virulence compared with e.g., *Salmonella* spp., co-infections with EAEC and an additional enteropathogen might be speculated to cause a stronger activation of the immune system. Co-infections with several enteric pathogens have previously been described to elicit a higher level of inflammation mediators (Qadri et al., 2002), which may result in faster eradication of enteropathogens. A large proportion of the patients investigated for EAEC suffered from traveler's diarrhea, which, in several studies, has proved a risk factor for infections with multiple pathogens (Paschke et al., 2010; Lääveri et al., 2014). In our study, 265 of 287 patients (92%) suffered from travelers' diarrhea, and 48 were seen to be infected with EAEC and an additional microorganism.

Totally, 135 clinical EAEC strains causing either STD or LTD were examined by a multiplex PCR for the presence of virulence genes. The most frequently detected virulence genes were *aggR* (transcriptional activator) and *aap* (dispersin protein) at 97.8%. Compared with other studies (Jiang et al., 2002; Kahali et al., 2004), we detected a higher frequency of these genes, which could have two explanations: First, working with an optimized PCR protocol allowing for better detection of EAEC genes. Secondly, we required the presence of at least two EAEC genes, potentially introducing strain selection bias. Of the AAFs, we found that AAF/I (*aagA*) was the most prevalent at 22.2%, which corresponds well with other studies (Boisen et al., 2012; Jønsson et al., 2015). Of the toxin genes, we found *pic* to have the highest prevalence (75.6%), followed by *astA* (34.8%) and *sepA* (34.1%). A study by Boisen et al. found the *sat* gene to be the single-most common gene (74.5%) in a large EAEC strain collection from multiple countries. However, *pic* was detected in 63.6% of the EAEC strains, and *sepA* in 38.2% of the EAEC strains (Boisen et al., 2009). The SPATEs are extracellular proteases secreted by bacteria and are thought to play an important role in secretory diarrhea during EAEC infection (Hebbelstrup Jensen et al., 2014). Therefore, it is not surprising that we observe a high frequency of toxins in strains isolated from patients suffering from an EAEC infection.

We assessed combinations of putative virulence factors by employing the CART analysis, and this suggested that the toxin *astA* was the virulence gene most strongly associated with LTD, regardless of any of the other 16 genes scored. The *astA* gene encodes the enterotoxin EAST-1, which is commonly detected in EAEC strains, and has previously been associated with watery diarrhea (Ménard and Daniel Dubreuil, 2002; Kaper et al., 2004). Previously, we have described how the *astA* is important in prolonged diarrhea in children (Hebbelstrup Jensen et al., 2017). However, many commensal *E. coli* strains also harbor the EAST-1 toxin, and it has, therefore, been suggested that additional virulence factors are required to mediate diarrhea (Konno et al., 2012). Among the *astA*-negative strains, it appeared that having a VFS > 9 could be associated with persistent diarrhea, whereas a VFS < 9 required the toxins *sigA* and *pet* for an association with LTD. Overall, our CART analysis does not provide a clear picture of a gene combination associated with LTD, but the importance of the toxin genes and a high VFS were observed, which correlates with the general belief that multiple virulence factors are important for EAEC pathogenesis (Hebbelstrup Jensen et al., 2014).

We detected high levels of antibiotic resistance among 137 EAEC strains isolated from Danish adults suffering from diarrhea. Previously, we have detected MDR among 35% of EAEC strains in Danish children in daycare (Hebbelstrup Jensen et al., 2016), and in 38% of EAEC strains isolated from Danish children with diarrhea (Hebbelstrup Jensen et al., 2017). Surprisingly, in this study, we detected a high level of resistance toward ciprofloxacin (34%), which is still one of the most frequently used antimicrobials for diarrhea (Kong et al., 2015). In earlier studies, EAEC was described as usually being susceptible to ciprofloxacin (Okeke and Nataro, 2001), and this increase in resistance is a serious cause for concern and needs to be considered when treating cases of travelers' diarrhea. In our study period from 2011 to 2014, the ciprofloxacin-resistance levels in invasive *E. coli* isolates from patients in Denmark were stable between 12 and 14%. The same tendency was observed for *E. coli* urine isolates from patients in Danish hospitals (Borck Høg et al., 2016). This could indicate that there is a high antibiotic resistance in EAEC isolates as well. However, we also detected a high degree of MDR EAEC strains (58%), and the majority of the strains were isolated from patients with reports of foreign travel (88%), indicating that we might be introducing MDR strains into the Danish population when returning from traveling, especially in Asia and Africa. A Spanish study from 2009 found high levels of resistance in EAEC strains isolated from travelers. In particular, they report a high percentage of resistance to quinolones in EAEC strains isolated from travelers to North Africa and India. They also reported an increase in antibiotic resistance between two time periods (1994–97 and 2001–04) (Mendez Arancibia et al., 2009). The importance of the MDR EAEC strains has been reflected in a community outbreak of urinary tract infection in Denmark by ST10 O78:H10 EAEC clone (Olesen et al., 2012; Boll et al., 2013).

The high number of MDR EAEC strains detected in our study is a cause for concern. A high number of MDR EAEC strains has previously been reported from studies conducted in Asia (75.8%) (Chattaway et al., 2017) and Africa (61%) (Seidman et al., 2016), but, as seen in our study, it also comprises industrialized countries. Colonization with bacterial strains with a high level of resistance in the intestinal tract of children, who are naïve to antibiotic treatment, was discovered (Sydenham et al., 2017). This indicates a considerable sharing of enteric microorganisms in the community (Johnson et al., 2008), where a host susceptible to EAEC infection can have very limited treatment options. Host genetic susceptibility to acquire an EAEC infection has been discussed previously (Jiang et al., 2003; Mohamed et al., 2011), and a high rate of asymptomatic carriage of EAEC was reported in other studies (Nüesch-Inderbinen et al., 2013). As EAEC strains are believed to consist of pathogenic as well as nonpathogenic strains (Hebbelstrup Jensen et al., 2014), the clinical decision whether to treat a person or not with EAEC is difficult. In addition, the clinicians may be forced to submit the patient suffering from diarrhea to additional medical examinations to diagnose the cause of diarrhea, since the individual pathogenic significance of EAEC continues to be discussed. Ciprofloxacin was ineffective toward limiting the duration of diarrhea in patients in our study. Although this drug has previously shown to have a therapeutic effect in EAEC-associated diarrhea (Glandt et al., 1999), it is not recommendable for treating EAEC infection, especially considering the high level of resistance in travelers' diarrhea.

## Conclusion

The EAEC was detected only in two out of 158 healthy adult controls (1.2%), and is, therefore, not expected to be common in the healthy Danish population. The majority of EAEC-infected patients suffered from long-lasting diarrhea >14 days and by follow-up, we discovered that 16 patients had undergone invasive examinations with e.g., endoscopy for examinations of the cause of diarrhea with no signs of pathology. The *astA* gene and a high VFS were both found to be important in the development of long-lasting diarrhea. Multidrug resistance in EAEC strains was found in a surprisingly high number in Danish patients suffering from diarrhea. Assessing the types of treatment and effect of treatment on duration of diarrhea, it was seen that antibiotics, especially ciprofloxacin, was ineffective toward limiting the duration of diarrhea in EAEC cases. Therefore, it is suggested that antibiotics should be used with care only in predisposed individuals or in patients with severe comorbidity.

## Author contributions

BH and CA contributed equally to this article and share first authorship. BH interpreted data and drafted the article. CA, conducted experimental work, interpreted data and drafted the article. SH provided statistical analysis. DR, AF-M, AH, LP, and JE collected samples. HM-L established the clinical database. CS and RP conducted experimental work. AP, JE, and KK oversaw the funding efforts. All authors approved the final draft and take responsibility for the integrity and the accuracy of this research and the interpretation hereof.

### Conflict of interest statement

The authors declare that the research was conducted in the absence of any commercial or financial relationships that could be construed as a potential conflict of interest.
